# Ophthalmology and Otology

**Published:** 1897-12

**Authors:** 


### SELECTIONS AND ABSTRACTS.

### OPHTHALMOLOGY AND OTOLOGY.

EDITED BY ALEX. W. STIRLING, M.D.

### OTORRHEA AND LIFE INSURANCE.

The great questions with insurance companies is: Why cannot a person with chronic inflammation of the middle ear be considered a good risk? Dr. D. B. St. John Roosa, in his treatise on "Diseases of the Ear," says: "The almost inevitable consequences of chronic suppuration of the middle ear are dangerous to the health and life of the patient, hence the importance of the subject and the interest which every physician should take in arresting the disease. It is in view of these facts, in regard to chronic suppuration of the middle ear, that English life insurance companies (as well as American) are said to decline to insure the lives of persons who are affected with the disease. A little consideration will show that any person who has a hole in the tympanic membrane and an ulcerative process in the parts beyond has a much less chance of having long life than one whose brain and vascular circulation are not thus exposed to the ravages of the disease. *There are very few persons, comparatively, who suffer from chronic suppuration and live out their days, while many of them die very young.*"

We have just received a copy of the work of Seth Scott Bishop, M.D., of Chicago. Speaking of the prognosis of chronic suppurative inflammation of the middle ear, he says: "This is a progressively destructive disease. Its tendency is not to spontaneous resolution; while many attacks may appear to get well of themselves, so long as the diseased condition remains, just so long recurring attacks will succeed each other. With every fresh cold, back comes the flux. The disease continues, though no discharge may make its appearance, and the patient is lulled into a false sense of security. A slight exciting cause sets up another exacerbation of the existing inflammation. Moreover, the natural tendency of his trouble is towards the bone. The mucous membrane of the middle ear answers the purpose of a periosteum, and the intimate relation of these structures jeopardizes the integrity of the osseous tissues when destructive processes are going on in the membranous lining. It has also been shown that mastoid suppuration is an offspring of the middle ear inflammation. The same may be said of phlebitis and sinus-thrombosis, meningitis, subdural abscess, pyemia and abscess of the brain. Only with proper treatment is the prognosis good."

A patient who has chronic inflammation of the middle ear is, therefore, always in danger of dying from its consequences, sooner or later. This is stated very emphatically by aurists, as we have seen. These consequences may exhibit themselves in various directions, as, for instance, inflammation of the mastoid cells. The mastoid is a part of the temporal bone, and is the prominent bone which is felt behind the ear. It is full of cells lined with mucous membrane and connected with the inner ear, so that inflammation of the ear can readily find its way into these cells, and frequently they are bathed in pus. The position of the patient when lying on his back assists the flow of pus into the mastoid cells. The inflammatory condition becomes so great that it overcomes the vitality of the patient. As a result the inflammation may extend to the bone itself, so that necrosis ensues. The labyrinth may become involved and as a further consequence we have blood poison or pyemia. The anatomical structure of the ear and contiguous organs is such that the inflammation may extend to the brain, forming abscesses, or to the covering or meninges of the brain, causing meningitis; and as a further consequence we may have paralysis, either of the face or of the brain itself. Besides, there are polypous growths from the tympanum, or from that part of it which is left, for there is always, as a rule, perforation or loss of the tympanum, or a part of it.

It is the opinion of some examiners that so long as the tympanum is perforated, thus permitting the pus to discharge freely, there is comparatively little danger, and therefore it is safe to issue a modified form of policy. It is, however, the province of the examiner to gather and report the facts. It is the office of the medical director to accept or reject upon these facts.

Strictly speaking, however, as "this is a destructive disease," we should be extremely cautious about accepting applicants who have otorrhea, even if there is a free opening through the tympanum, no discharge visible, or if the case is non-progressive. There is always danger of an exacerbation, and of a rapidly fatal termination. From an insurance point of view it is bad judgment to accept such cases on any terms. Personally we are of the opinion that these cases should be rejected.—*Dr. G. W. Wells, Medical Examiner*.

---

### FORMALIN IN OPHTHALMOLOGY.

Of the remedies that have recently attracted attention in ophthalmological therapeutics, formic aldehyde is probably the most important. The preparation used is that known as FORMALIN. It is a forty per cent. solution of formic aldehyde. The solutions mentioned below are dilutions of formalin. Guaita (*Ann. d'Ottal.*, v. xxiii, p. 360) was one of the first to exploit the excellencies of this remedy in ophthalmic practice. The immediate germicidal power of formic aldehyde is much inferior to solutions of the sublimate of similar strength, but its great diffusibility, due to the absence of any tendency to coagulate albumin, renders it of greater value as an antiseptic. Cutting instruments are not affected by it. Guaita found formalin of value in the treatment of corneal ulcers, infected wounds, purulent and mucopurulent conjunctivitis, and trachoma. He employed a solution of 1–100 to touch the corneal ulcer, and gave a solution of 1–2000 to be used as a collyrium every four hours. Formalin in solution of 1–1000 or less is somewhat irritating to the conjunctiva, but in solution of 1–2000 it produces no unpleasant sensation; in this strength the remedy is harmless. Valude (*Recueil d'Ophthal.*, 1893, p. 431) substantiates the claims regarding the value of formic aldehyde as set forth by Guaita; he has found it efficacious in post-operative infection, instilling a solution of 1–1000 into the eye every three hours. Davidson (*British Med. Jour.*, 1896, p. 144) uses a solution of formalin (Schering) 1–2000 to 1–3000, directing that it shall be dropped into the eye freely every hour—a virtual lavage of the eye. Employed in this way it has proven to be of great value in cases of infected corneal ulcer, with or without hypopyon. One of the cases reported by Davidson is briefly as follows: Prolapse of the iris accompanying a perforating lacerated wound of the cornea, in its lower half, incurred four days previously. Status praesens: Infiltration of corneal tissue adjacent to the wound; hypopyon; iritis. Treatment: atropin, one per cent. instilled two or three times daily. Eight or ten drops of a 1–2000 solution of formalin was dropped into the eye every hour when the patient was awake. When seen the next day the cornea was clear, hypopyon gone, iris fairly well dilated, no pain. Burnett (*Ophthalmic Record,* 1896, p. 310) has met with much success in the use of formic aldehyde in the treatment of corneal ulcers, muco-purulent and purulent conjunctivitis. He employs the Schering preparation in the strength of 1–1000 to 1–2000, applied every four hours.—*Dr. J. E. Weeks, in Medical News.*

---

### SOME EXPERIMENTS ON THE UNION OF CORNEAL WOUNDS.

Mr. Ernest Clarke related experiments consisting of various operations on the cornea of rabbits whereby the anterior chamber was completely emptied of aqueous with the view of ascertaining the time taken by these wounds in uniting sufficiently to allow the anterior chamber to be reformed. Description of the methods adopted in operating and preserving the specimens were given. Two classes of experiments were performed. In the first class the animal was kept under an anesthetic and killed. The anterior chamber was found to be present in two minutes, and fully reformed in twenty-five minutes. In the second class the animal was allowed to recover, and, after varying intervals, ranging from half an hour to two hours and a-half, again placed under an anesthetic and killed. The movements of the animal caused delay in the reformation of the anterior chamber. The average time was an hour and a half. Wounds at the upper margin united most rapidly with less scar. Wounds across the center of the cornea and at the lower margin took longer to repair and made a larger scar. The rapidity with which the anterior chamber was reformed suggested that if, during an operation on the eye where the presence of the aqueous was necessary, this aqueous were accidentally lost, the eye should be bandaged, and the continuance of the operation postponed only for half an hour at the outside instead of postponing it until the next day, as was generally done. The experiments also showed the great influence that rest had on the process of repair, and emphasized the importance of keeping patients absolutely quiet for the first few hours after an eye operation.—*British Medical Journal.*

---

### TREATMENT OF STYES.

In the *Journal des Praticiens* the following treatment is suggested for this troublesome condition: Locally, as soon as the evidence of a stye is appearing, an attempt may be able to abort it by cauterizing the spot with the fine point of a galvano or thermo-cautery. In other instances, if it cannot be aborted it is best to aid the maturation of the boil by providing warmth and moisture, and evacuating the pus as soon as this is formed. In order to render the eyelid perfectly aseptic, it is well to wash the margins of the lid with one of the following solutions, hot:

| | |
|---|---|
| Bichlorid of mercury | 4 grs. |
| Distilled water | 1 pt. |

Or, in place of this, if it is thought the individual will be susceptible to the action of the mercury:

| | |
|---|---|
| Bichlorid of mercury | 2 grs. |
| Distilled water | 1 pt. |

In other instances an ointment made as follows is of value:

| | |
|---|---|
| Powdered calomel | 4 grs. |
| Vaselin | 80 grs. |

As a general rule, styes tend to return, owing to auto-inoculation. Care, therefore, should be taken that the edges of the lids are kept well cleansed, and if necessary a mild antiseptic wash should be used for some time after one stye has healed, in order to prevent the coming of others. Careful attention should also be paid to the condition of the alimentary canal. Or, if the patient is young, with scrofulous, arthritic or anemic tendencies, Fowler's solution in full doses may be administered with great advantage.—*Therapeutic Gazette.*

---

TREATMENT OF OPHTHALMIA NEONATORUM.

The first evidence of the disease is a tear-drop in the inner canthus of the affected eye. Usually but one eye is affected at a time. This glairy fluid, for it is not a natural secretion, if examined closely is very much like mucilage of gum arabic, and appears during the first twenty-four hours. At this time nitrate of silver solution, five grains to the ounce, should be applied to the everted eyelids once daily. At the end of the second day this sticky fluid has changed its consistency and appearance, and it has become muco-purulent.

When the disease has developed the best treatment is the application of a five-grain solution of silver to the conjunctiva of the lids, and this followed during the day with sublimate solution of 1 to 5,000. Whenever pus makes its appearance let it be wiped away from between the lids with a pellet of absorbent cotton saturated with the sublimate solution. The edges of the lids may be anointed with ointment of yellow oxide of mercury, an eighth of a grain to the dram, to which has also been added corrosive sublimate, grain 1 to 3,000.

If the flow of pus is not lessened by this treatment it may be checked by applying diluted (one-half) peroxide of hydrogen. It is important to irrigate the cul-de-sac up and about the retrotarsal fold, which is best done with a hydrostatic eye douche. In this stage, after there has been a discharge of pus for four or five days, and, owing to the tumefaction of the eyelids, which has caused a disturbance of the circulation and nourishment of the cornea, a haziness appears, then apply a solution of eserine sulph., grain one-fourth to dram, in addition to the treatment already inaugurated.

In the second stage, when the conjunctiva is thick and velvety, apply a stronger silver solution. One may even scarify the conjunctiva and apply the sulpho-carbolate of zinc lotion. In the third stage apply the silver solution (five grains to one ounce)

once daily and bathe frequently. By following out Crede's method in the beginning, and this line of treatment, blindness from this disease will be lessened to almost nothing. In the absence of nitrate of silver any astringent wash is better than nothing; alum, always on hand, will be of some value, and in the absence of any astringent, plenty of water with a pinch of soda bicarb., to dilute the pus and wash away the micrococci, is of some value.—*Dr. L. Webster Fox, Medical Council.*

---

### OPERATIVE TREATMENT OF PTERYGIUM.

Dr. Edouard Boeckmann, St. Paul, suggested an operation which was a combination of some others referred to. A crescentric piece is cut from the pterygium about five lines from its head. This part is curetted thoroughly down to the sclerotic. The head of the pterygium is dissected off. At the convexity of the piece cut out a stitch is inserted and the opposing edges drawn together. This leaves the curetted portion to granulate and form a cicatrix. The author thinks the results from this method superior to that of any other in his experience. The paper was discussed by Drs. Wilder and Buckner.—*Ex.*

---

### ICHTHYOL IN AFFECTION OF THE EYES.

Germani (*Gazetta degli ospedali*, June 20, 1896; *British Medical Journal*, August 22, 1896) finds that lanolin mixed with from ten to fifteen per cent. of its weight of ichthyol is very efficacious in ciliary blepharitis, curing it when the ordinary yellow ointment has failed. Collyria containing from one to three per cent. of ichthyol are very useful in phlyctenular conjunctivitis and in simple catarrhal ophthalmia. Ichthyol is well borne, soon eases the pain, and hastens the cure.—*N. Y. Med. Jour.*

